# Chemical Composition and Antimicrobial Activity of the Essential Oils from Three Chemotypes of *Origanum vulgare* L. ssp. *hirtum* (Link) Ietswaart Growing Wild in Campania (Southern Italy)

**DOI:** 10.3390/molecules14082735

**Published:** 2009-07-27

**Authors:** Laura De Martino, Vincenzo De Feo, Carmen Formisano, Enrico Mignola, Felice Senatore

**Affiliations:** 1 Dipartimento di Scienze Farmaceutiche, Università degli Studi di Salerno, via Ponte don Melillo, 84084 Fisciano (Salerno), Italia; E-mail: defeo@unisa.it (V.D.F.); 2 Dipartimento di Chimica delle Sostanze Naturali, Università degli Studi di Napoli, “Federico II”, Via D. Montesano, 49, 80131 Napoli, Italia; E-mails: caformis@unina.it (C.F.), mignolaenrico@alice.it (E.M.), fesenato@unina.it (F.S.)

**Keywords:** *Origanum vulgare* ssp. *hirtum*, essential oil composition, thymol, carvacrol, linalyl acetate, antibacterial activity

## Abstract

Essential oils obtained from inflorescences of three *Origanum vulgare* L. ssp. *hirtum* (Link) Ietswaart samples, growing wild in different locations in Campania (Southern Italy), were analysed. Three chemotypes were found: the first, with a prevalence of carvacrol/thymol; the second, characterized by the prevalence of thymol/α-terpineol; the third, featuring a prevalence of linalyl acetate and linalool. This chemical study attempts to provide a contribution in shedding light on the relationship between chemical composition and biotypes and/or chemotypes in *Origanum vulgare* ssp. *hirtum*. The essential oils were also evaluated for their antibacterial activity against 10 selected microorganisms. The data obtained contribute to the future view to use the essential oils as natural preservatives for food products, due to their positive effect on their safety and shelf life.

## Introduction

Many different species, commonly known as *oregano* or *origanum*, are of economic interest, although they belong to different botanical families and genera. Four main groups commonly used for culinary purposes can be distinguished, i.e., Greek oregano (*Origanum vulgare* L. ssp. *hirtum* (Link) Ietswaart), Spanish oregano (*Coridothymus capitatus* (L.) Hoffmanns & Link), Turkish oregano (*Origanum onites* L.) and Mexican oregano (*Lippia graveolens* HBK) [1,2,3]. In Europe and, in general, all over the world, the most commonly found oregano species belong to the botanical genus *Origanum*. Within this genus, Ietswaart [4], based on morphological criteria, recognised three groups, 10 sections, 38 species, six subspecies and 17 hybrids. Before 1980, *O. vulgare* L. referred indifferently the subspecies that Ietswaart later identified as *O. vulgare* L. ssp. *hirtum* (Link) Ietswaart, *O. vulgare* L. ssp. *gracile* (C. Koch) Ietswaart, *O. vulgare* L. ssp. *vulgare*, and *O. vulgare* L. ssp. *viride* (Boiss.) Hayek. The *O. vulgare* subspecies are not easily distinguishable by their morphological aspects alone. It is not surprising that many analytical investigations about oregano essential oils did not discriminate between the numerous subspecies that show subtle morphological and chemical differences. The characteristics which seem to be quite constant are the yield and the composition of their essential oils, thus being these characteristics useful for subspecies identification. *O. vulgare* L. ssp. *vulgar*e and *O. vulgare* ssp. *hirtum* (Link) Ietswaart are commercial species. A few references have reported on the first species [5,6,7,8], while more numerous works have dealt with the second [8,9,10,11,12,13,14], even if the reported data can vary widely probably depending on the different growing conditions and geographical areas in which the analyzed plants were collected [2]. *Origanum vulgare* L. ssp. *hirtum* (Link) Ietswaart is a typical East Mediterranean taxon. Ecologically, this species prefers warm, sunny habitats and loose, often rocky, calcareous soils, usually low in moisture content. Though very variable in morphological aspects, it can be distinguished from other *O. vulgare* subspecies by its hairy stems, compact inflorescences, leaves and calyces densely covered with glandular structures, green bracts, which are usually as long as calyces, and white flowers [15]. A number of studies have shown that *O. vulgare* ssp. *hirtum* is a very variable taxon both in morphological and in chemical features [16], with an essential oil whose principal components are phenols, *p*-cymene, and γ-terpinene [2]. As a continuation of our research on the oils of the Lamiaceae growing wild in Southern Italy [17,18,19,20,21,22], in this work we examined the composition of the essential oils of three *O. vulgare* L. ssp. *hirtum* populations growing wild in Campania (Southern Italy) and their antimicrobial activity on ten selected microorganism. *O. vulgare* ssp. *hirtum* is largely employed as an aromatizer in both traditional and modern foods, namely in Southern part of Italy. Its use is very old and this plant is often collected from wild populations, dried and then preserved in kitchen cupboards.

## Results and Discussion

### Chemical composition of the essential oils

The isolated oils were dried over anhydrous sodium sulphate and stored at 4-6 °C under N_2_. The dry materials gave yellow-reddish oils in a yield 2.35% (w/w, F), 3.15% (w/w, S), and 2.93% (w/w, SG) of essential oil, characterized by a typical odour.

Table 1 shows the relative percentage of the volatile components identified in the oils; compounds are listed according to their linear retention indices (LRIs) on a HP 5MS column. A total of 64 compounds have been identified in the three oils. The essential oil composition of the three populations of plants appeared quite different and allows us to identify three different chemotypes. In fact, the first and second oil were characterized by high percentages of phenols, but while the Furore oil (F) can be classified as a carvacrol/thymol chemotype, the Sanza sample (S) can be classified as a thymol/α-terpineol chemotype. The oil from San Giovanni a Piro (SG) could be classified as a linalyl acetate/linalool chemotype, with these compounds accounting for 15.90% and 12.50%, respectively. In F, the total phenol content represents 45.70% of the oil with comparable percentages of carvacrol, thymol and their derivatives (23.34% and 22.16%, respectively). In S, total phenols represent 40.50% of the oil with a prevalence of thymol and its derivatives (29.33%) while in SG the phenolic fraction accounted to 6.30% of the oil, the half of which is represented by thymol (3.24%). Monoterpene hydrocarbons were present in different amounts: 11.60%, 15.60% and 26.30%, respectively for the F, S and SG oil, but in all cases γ-terpinene and *p*-cymene were the most abundant. γ-Terpinene and *p*-cymene, the biosynthetic precursors of monoterpenoid phenols, were present in the same amount in the oil F (2.38% and 2.81%, respectively), while in the oil S γ-terpinene (4.59%) was detected in a higher percentage than in the oil F, but it wasn’t the same for *p*-cymene (1.25%). However, in both cases, the two monoterpene hydrocarbons, γ-terpinene and *p*-cymene, were constantly present in the essential oils analysed, but always in lower amounts than those of the phenols, according to the results described by a previous paper [17]. The oxygenated monoterpenes represent 5.60% (F), 21.60% (S) and 33.70% (SG) of the oils. In the oils were totally detected 22 sesquiterpene hydrocarbons that ranged between 21.60% (F) and 12.70% (S). In all oils, γ-muurolene (4.48%-2.59%), (E)-β-caryophyllene (4.29%-2.11%), β-bisabolene (4.13%-2.51%) and δ-cadinene (3.17%-0.09%) were the most abundant sesquiterpenes of this fraction being the other components present in lower amounts, traces or absent. Oxigenated sesquiterpenes the most abundant were α-cadinol (4.02%-1.06%) and spathulenol (3.90%-1.20%).

The available literature [2] reports the presence of a carvacrol/thymol chemotype of *O. vulgare* ssp. *hirtum*; the others two chemotypes were described for the first time. Some papers describe the essential oil composition of *O. vulgare* ssp. *hirtum* from different geographic areas. Russo *et al*. [2] reported four chemotypes for this species, growing in Calabria (Southern Italy), on the basis of their phenolic content: thymol, carvacrol, thymol/carvacrol and carvacrol/thymol chemotypes, with the majority of samples belonging to a thymol chemotype. Kokkini *et al*. [16] studied the essential oils from *O. vulgare* ssp. *hirtum* plants collected in late autumn from six localities of three distinct geographic areas of Greece. They reported that oils of plants from the Northern part of Greece were rich in thymol, whereas those from the Southern part of the country were rich in carvacrol. Several samples rich in carvacrol were found also in Bulgaria [22].

Generally, chemotypes form “biochemical varieties” or “physiological forms” in botanical species, each of which with a specific enzymatic equipment. These species are genetically codified and direct their biosynthesis to the preferential formation of a definite compound. In the case of phenolic compounds, the metabolic pathway is through the autooxidative conversion of γ-terpinene to *p*-cymene followed by hydroxylation of *p*-cymene to thymol or carvacrol [23]. The phenols content, generally, is high during flowering stage in phenol-type origanum plant, as reported [24,25].

The characterization of habitat is of fundamental importance to understand species distribution. In a definite geographical area, the factors that weight heavily on chemotypes differentiation are mainly related to intrinsic factors such as sexual polymorphism or genetic mechanism, but for the phenolic essences, environmental conditions are able to influence biosynthetic pathway. At this regard, it is interesting note that the samples studied were collected in areas with different sun exposure. In fact, Furore is located in a sunny position, front the sea, Sanza is located in an internal zone, far from the sea, with temperatures lower than the other two places of sample collection, and San Giovanni a Piro is located quite near the sea. Also the season and the characteristic of plant (fresh or dried) can determinate noticeable differences in the total oil content and the concentration of the main oil components: in particular, the proportion of carvacrol has been shown to be much higher in the summer; in the autumn, *p*-cymene predominates [16,17,26].

Very few papers have reported an oregano chemotype characterized by the presence of linalyl acetate/linalool but never in *O. vulgare* ssp. *hirtum*. In fact, Perez *et al*. [27] reported linalool as the main volatile component of *Origanum vulgare* ssp. *virens*, characterized by a high quantity of linalool and a low quantity of thymol. D’Antuono *et al*. [28] reported a Northern Italian population of *O. vulgare* L. rich in linalool. On the other hand, Mockute *et al*. [29] showed that the main constituents of the essential oil of *O. vulgare* ssp. *viride*, wild in Iran, were linalyl acetate, β-caryophyllene and sabinene. Data reported in this work should help to throw light in the apparent complex chemotaxonomy of the genus *Origanum*.

### Antimicrobial activity

The Minimum Inhibitory Concentration (MIC) and the Minimum Bacterial Concentration MBC values of the essential oils against 10 selected microorganisms are reported in Table 2. The essential oils showed action mainly against the Gram-positive pathogens, among which *S. epidermidis* was the most affected. Among Gram-negative bacteria, only *E. coli* was affected by the oil F. The oil F and S resulted more active than oil SG and presumably this activity is related to phenolic components, such as thymol, carvacrol, carvacrol methyl ether, though part of the activity could result from the synergistic presence of minor active constituents, such as γ-terpinene and *p*-cymene.

The antibacterial activity results seem to be in accordance with previous reports indicating that the essential oils, rich in phenolic compounds, possess high levels of antimicrobial activity [30,31,32,33]. Loźienė *et al*., [34] have reported the antimicrobial activity of phenolic compounds, such as carvacrol and thymol. It is noteworthy that has been suggested that phenolic derivatives can cause membrane-disrupting activities [35]. Linalol proved to be a very active compound: in fact, Mazzanti *et al*., [36] reported that such component inhibited the growth of different microorganism; linalyl acetate showed bacteriostatic activity [37]. The lower antimicrobial activity of the oil SG should also be due to a synergistic presence of minor active constituents, such as α-terpineol, thymol, γ-terpinene and *p*-cymene [38,39,40,41,42,43].

## Experimental

### Plant material

Samples of *Origanum vulgare* L. ssp. *hirtum* (Link) Ietswaart were collected from populations growing wild in different areas of Salerno province (Campania, Southern Italy) in June 2008: Furore, 600 m s. l. (F), Sanza, 500 m s. l. (S) and San Giovanni a Piro, 450 m s. l. (SG). Voucher of each sample were stored in the Herbarium of the Salerno University: Sa 05/08, Sa 06/08 and Sa 07/08, respectively for F, S and SG. The representative homogeneous sample of each population was collected during “balsamic time” corresponding to the flowering stage. The plant material used for the isolation of the essential oil was air-dried at room temperature.

### Oil extraction

Lots of twenty grams of dried inflorescences were hydrodistilled for three hours in a Clevenger type apparatus, as previously described [44].

### Gas Chromatography

Gas Chromatography (GC) analyses were carried out using a Perkin-Elmer Sigma-115 gas chromatograph equipped with a data handling system and a flame ionization detector (FID). Separation was achieved by a fused-silica capillary column HP 5MS, 30 m length, 0.25 mm internal diameter, 0.25 µm film thickness. The operating conditions were as follows: injector and detector temperatures, 250 °C and 280 °C, respectively; oven temperature programme: 5 min isothermal at 40 °C, subsequently at 2 °C/min up to 250 °C and finally raised to 270 °C at 10 °C/min. Helium was used as the carrier gas (1 mL/min). Diluted samples (1/100 v/v, in *n*-pentane) of 1 µL were manually injected at 250 °C, and in the splitless mode. Analysis was also made by using a fused silica HP Innowax polyethylenglycol capillary column (50 m x 0.20 mm i.d.; 0.20 µm film thickness). The percentage composition of the oils was computed by the normalization method from the GC peak areas. The analysis have been carried out in triplicate and the results are expressed as mean ± SD.

### Gas Chromatography - Mass Spectrometry

Gas Chromatography-Mass Spectrometry (GC-MS) analysis was performed using an Agilent 6850 Ser. A apparatus, equipped with a fused silica HP-5 capillary column (30 m x 0.25 mm i.d.; film thickness 0.33 µm), linked on line with an Agilent MSD 5973 Mass Selective Detector; ionization energy 70 eV, multiplier voltage 2000 V. Mass spectra were scanned in the range 35-450 amu, scan time 5 scans/s. Gas chromatographic conditions were as given above, transfer line was kept at 295 °C. The oil components were identified from their GC retention indices, with either those of the literature [45,46] or with those of authentic compounds available in our laboratories. The identity of the components was assigned by comparing their linear retention indices, relative to C_8_-C_28_ *n*-alkanes, under the same operating conditions. Further identification was made by comparison of their MS spectra on both columns, with either stored in NIST 02 and Wiley 275 libraries or with mass spectra from the literature [45,46,47] and our homemade library. The analyses were carried out in triplicate and the results are expressed as mean ± SD.

### Antibacterial activity

The antibacterial activity was evaluated by determining the minimum inhibitory concentration (MIC) and the minimum bactericidal concentration (MBC), using the broth dilution method [48,49,50]. Ten bacterial species, selected as representative of the Gram+ and Gram- classes, were tested: *Bacillus cereus* (ATCC 11778), *Bacillus subtilis* (ATCC 6633), *Staphylococcus aureus* (ATCC 25923), *Staphylococcus epidermidis* (ATCC 12228)*, Streptococcus faecalis* (ATTC 29212), *Escherichia coli* (ATCC 25922), *Proteus mirabilis* (ATCC 25933), *Proteus vulgaris* (ATCC 13315), *Pseudomonas aeruginosa* (ATCC 27853), *Salmonella typhi* Ty2 (ATCC 19430). The strains were maintained on Tryptone Soya agar (Oxoid, Milan, Italy); for the antimicrobial tests, Tryptone Soya broth (Oxoid, Milan, Italy) was used. In order to facilitate the dispersion of the oil in the aqueous nutrient medium, it was diluted with Tween 20 at a concentration of 10%. Each strain was tested with sample that was serially diluted in broth to obtain concentrations ranging from 100 µg/mL to 0.8 µg/mL. The sample was previously sterilized with a 0.20 µm Millipore filter. The sample was stirred, inoculated with 50 µL of physiological solution containing 5 x 10^6^ microbial cells, and incubated for 24 h at 37 °C. The MIC value was determined as the lowest concentration of the sample that not permit any visible growth of the tested microorganism after incubation. Control, containing only Tween 20 instead of the essential oil, was not toxic to the microorganisms. Cultures, containing only sterile physiologic solution Tris buffer, were used as positive control. MBC was determined by subculture of the tubes with inhibition in 5 mL of sterile nutrient broth. After incubation at 37 °C the tubes were observed. When the germs don’t grow, the sample denoted a bactericidal action. Oil samples were tested in triplicate and the experiment was performed three times. The results are expressed as mean ± SD. Gentamycine was used as reference agent.

## Conclusions

Essential oils are presently regarded as useful in food technology [51], in fact, their use in food preparation accomplishes both food flavouring and food preservation. Data obtained clearly showed the inhibitory activity of the essential oils tested against pathogenic bacteria. The available literature [52,53] shows that essential oils are natural preservatives in food and/or pharmaceutical industry: this use represents a viable and safe way to decrease the utilisation of synthetic food preservatives, due to their positive effects both on safety and shelf life.

## Figures and Tables

**Table 1:** Essential oil composition (% of total) of aerial parts of *O. vulgare* ssp. *hirtum* (Lamiaceae) growing wild in Southern Italy.

K_i_^a^	K_i_^b^	Compound	Identification^c^	F^d^	S^d^	SG^d^
925	1013	Tricyclene	LRI, MS	0.29±0.02	0.20±0.01	0.10±0.01
928	1035	α-Thujene	LRI, MS	0.11±0.01	0.28±0.01	0.21±0.01
938	1075	α-Pinene	LRI, MS, Co-GC	0.19±0.03	0.42±0.01	0.19±0.02
945	1056	Camphene	LRI, MS, Co-GC	0.50±0.02	t	0.22±0.01
973	1132	Sabinene	LRI, MS, Co-GC	0.20±0.01	0.28±0.04	0.88±0.03
978	1118	β-Pinene	LRI, MS, Co-GC	0.11±0.01	0.32±0.03	0.40±0.01
980	1154	1-Octen-3-ol	LRI, MS	0.30±0.04	0.30±0.01	
983	1253	Octan-3-one	LRI, MS	t	0.10±0.01	
993	1173	Myrcene	LRI, MS, Co-GC	0.70±0.09	0.90±0.06	2.80±0.20
1001	1146	δ^2^-Carene	LRI, MS	0.11±0.01	0.30±0.02	0.22±0.01
1005	1150	α-Phellandrene	LRI, MS, Co-GC	0.29±0.02	0.50±0.03	0.08±0.00
1008	1160	δ^3^-Carene	LRI, MS	0.27±0.02	1.10±0.10	0.19±0.02
1013	1189	α-Terpinene	LRI, MS, Co-GC	0.33±0.03	0.50±0.03	0.41±0.03
1025	1278	*p*-Cymene	LRI, MS, Co-GC	2.81±0.20	1.25±0.3	2.01±0.10
1029	1218	β-Phellandrene	LRI, MS, Co-GC	0.19±0.02	0.15±0.00	0.09±0.01
1030	1205	Limonene	LRI, MS, Co-GC	0.27±0.01	1.21±0.3	2.36±0.20
1034	1213	1,8-Cineole	LRI, MS, Co-GC	0.50±0.03	0.50±0.04	0.60±0.03
1038	1243	(*Z*)-β-Ocimene	LRI, MS	0.83±0.04	0.69±0.01	4.64±0.40
1049	1262	(*E*)-β-Ocimene	LRI, MS	0.22±0.01	1.71±0.30	4.10±0.20
1057	1256	γ-Terpinene	LRI, MS, Co-GC	2.38±0.40	4.59±0.80	4.90±0.10
1086	1265	Terpinolene	LRI, MS	1.80±0.5	1.20±0.1	0.50±0.03
1097	1553	Linalool	LRI, MS, Co-GC	2.87±0.30	4.10±0.2	12.50±0.7
1128	1638	*cis*-*p*-Menth-2-en-1-ol	LRI, MS	0.13±0.01	t	
1167	1718	Borneol	LRI, MS, Co-GC	0.33±0.01	0.29±0.02	t
1176	1611	Terpinen-4-ol	LRI, MS	1.07±0.20	0.41±0.01	0.80±0.01
1189	1706	α-Terpineol	LRI, MS	0.22±0.03	15.10±0.6	3.90±0.10
1239	1607	Thymol methyl ether	RI, MS, Co-GC	3.81±0.60	2.27±0.20	1.11±0.10
1245	1975	Carvacrol methyl ether	LRI, MS	1.19±0.40	4.63±0.10	0.79±0.03
1259	1665	Linalyl acetate	LRI, MS, Co-GC	0.48±0.04	1.20±0.1	15.90±0.5
1293	2198	Thymol	LRI, MS, Co-GC	18.21±0.80	26.75±0.70	3.24±0.30
1299	2239	Carvacrol	LRI, MS, Co-GC	21.89±0.70	6.45±0.65	0.46±0.02
1348	1466	α-Cubebene	RI, MS	0.20±0.01	0.10±0.01	0.10±0.00
1353	2186	Eugenol	RI, MS, Co-GC	0.20±0.02		
1356	1868	Thymyl acetate	LRI, MS	0.14±0.01	0.31±0.00	0.49±0.01
1367	1890	Carvacryl acetate	LRI, MS	0.26±0.02	0.09±0.02	0.21±0.02
1372	1493	α-Ylangene	LRI, MS	0.27±0.01	t	
1377	1497	α-Copaene	LRI, MS	0.29±0.03	0.15±0.01	
1382	1549	β-Cubebene	LRI, MS	0.21±0.04	0.25±0.02	0.10±0.02
1385	1535	β-Bourbonene	LRI, MS	0.33±0.05	0.20±0.01	0.60±0.01
1387	1600	β-Elemene	LRI, MS	0.10±0.01	0.10±0.00	0.20±0.00
1415	1612	(*E*)-β-Caryophyllene	LRI, MS	3.72±0.51	2.11±0.10	4.29±0.3
1432	1612	β-Gurjunene	LRI, MS	0.27±0.02	0.09±0.01	0.19±0.01
1432	1650	γ-Elemene	LRI, MS	0.29±0.03	0.22±0.02	0.15±0.01
1437	1628	Aromadendrene	LRI, MS	0.21±0.00	0.19±0.02	
1455	1689	α-Humulene	LRI, MS	1.71±0.20	1.48±0.20	1.35±0.20
1463	1662	*allo*-Aromadendrene	LRI, MS	0.19±0.03	0.11±0.00	0.50±0.04
1477	1726	Germacrene D	LRI, MS	0.13±0.00	0.41±0.02	2.11±0.30
1478	1704	γ-Muurolene	LRI, MS	4.48±0.58	2.59±0.20	3.61±0.20
1492	1756	Bicyclogermacrene	LRI, MS	0.35±0.06	0.13±0.03	
1494	1740	Valencene	LRI, MS	0.25±0.05	0.17±0.02	t
1503	1740	α-Muurolene	LRI, MS	0.22±0.01	0.20±0.01	0.39±0.01
1510	1743	β-Bisabolene	LRI, MS	4.13±0.42	2.81±0.20	2.51±0.20
1515	1776	γ-Cadinene	LRI, MS	0.81±0.06	0.29±0.01	0.35±0.01
1526	1773	δ-Cadinene	LRI, MS	3.17±0.51	0.99±0.1	2.09±0.30
1532	1745	α-Cadinene	LRI, MS	0.18±0.01	t	0.21±0.00
1544	1854	Germacrene B	LRI, MS	0.09±0.01	0.11±0.01	0.15±0.01
1565	2057	Ledol	LRI, MS	t	t	0.10±0.00
1577	1250	Spathulenol	LRI, MS	3.90±0.40	1.20±0.20	1.20±0.01
1579	2008	Caryophyllene oxide	LRI, MS	1.01±0.09	0.60±0.04	1.20±0.1
1636	2183	γ-Eudesmol	LRI, MS	0.21±0.03	0.14±0.00	0.38±0.03
1640	2158	t-Cadinol	LRI, MS	2.10±0.50	0.15±0.00	0.19±0.02
1642	2209	t-Muurolol	LRI, MS	t	0.05±0.01	1.51±0.20
1652	2235	α-Cadinol	LRI, MS	1.69±0.09	1.06±0.2	4.02±0.3
1668	2219	α-Bisabolol	LRI, MS	0.19±0.01	t	
		TOTAL		93.90	94.00	91.80

F: Furore sample; S: Sanza sample; SG: San Giovanni a Piro sample. The values are the mean of three replicates±SD. ^a^: HP 5MS column; ^b^: HP Innowax column ; ^c^: LRI = linear retention index, MS = mass spectrum, Co-GC = co-injection with authentic compound; ^d^: Mass of compounds in mg/100 mg oil; t, trace (<0.05%); mean value ± standard error, *n*, three independent determinations.

**Table 2:** MIC and MBC values (μg/mL) of essential oils from *Origanum vulgare* L. ssp. *hirtum* (Link) Ietswaart growing wild in Campania and MIC of reference antibiotic. Results are the mean of three experiments.

Bacterial strain	F MIC MBC	S MIC MBC	SG MIC MBC	G
*Bacillus cereus* ATCC 11778	50 50	50 50	50 100	1.56
*Bacillus subtilis* ATCC 6633	50 50	50 100	50 100	1.56
*Staphylococcus aureus* ATCC 2592	50 50	50 50	100	3.12
*Staphylococcus epidermidis* ATCC 12228	25 25	25 50	50 100	6.25
*Streptococcus faecalis* ATTC 29212	50 100	50 100	100 100	>100
*Escherichia coli* ATCC 25922	50 100	100 100	100 100	3.12
*Proteus mirabilis* ATCC 25933	100 100	100 100	>100	100
*Proteus vulgaris* ATCC 13315	100 >100	100 100	>100	100
*Pseudomonas aeruginosa* ATCC 27853	>100	>100	>100	12.5
*Salmonella typhi Ty2* ATCC 19430	100 100	100 100	>100	>100

F: Furore sample; S: Sanza sample; SG: San Giovanni a Piro sample; G = gentamycine.
